# Coagulase negative staphylococcal sepsis in neonates: do we need to adapt vancomycin dose or target?

**DOI:** 10.1186/s12887-016-0753-0

**Published:** 2016-12-08

**Authors:** Helgi Padari, Kersti Oselin, Tõnis Tasa, Tuuli Metsvaht, Krista Lõivukene, Irja Lutsar

**Affiliations:** 1 Pediatric and Neonatal Intensive Care Unit, Tartu University Hospital, 1a L. Puusepa street, 50406 Tartu, Estonia; 2 Hematology and Oncology Clinic, Tartu University Hospital, Tartu, Estonia; 3 Institute of Copmuter Science, University of Tartu, Tartu, Estonia; 4 Department of Clinical Microbiology, United Laboratories of Tartu University Hospital, Tartu, Estonia; 5 Institute of Microbiology, University of Tartu, Tartu, Estonia

**Keywords:** Vancomycin, Neonate, Pharmacokinetics, Population pharmacokinetics

## Abstract

**Background:**

Despite differences in types of infection and causative organisms, pharmacokinetic-pharmacodynamic (PKPD) targets of vancomycin therapy derived from adult studies are suggested for neonates. We aimed to identify doses needed for the attainment of AUC/MIC > 400 and AUC/MIC > 300 in neonates with sepsis and correlate these targets with recommended doses and treatment outcome.

**Methods:**

Neonates who had Vancomycin therapeutic drug monitoring (TDM) performed between January 1, 2010 and December 31, 2012 were studied. Clinical characteristics, episodes of Gram-positive sepsis with outcomes and all neonatal blood culture isolates in hospital were collected from medical records. To estimate probability of target attainment of AUC/MIC >400 and AUC/MIC >300 a 1000-subject Monte Carlo simulation was performed by calculating AUC using Anderson’s (Anderson et al. 2006) and TDM trough concentrations (C_trough_) based population PK models.

**Results:**

Final dataset included 76 patients; 57 with confirmed Gram-positive sepsis. TDM was taken after the 1^st^ to 44^th^ dose. 84.1% of C_trough_ were within the range 5–15 mg/L. Currently recommended doses achieved probability of the targets (PTA) of AUC/MIC >400 and AUC/MIC >300 in less than 25% and 40% of cases, respectively. Doses required for 80% PTA of AUC/MIC > 400 for MIC ≥2 mg/L resulted in C_trough_ values ≥14 mg/L. Mean AUC/MIC values were similar in treatment failure and success groups.

**Conclusion:**

With currently recommended vancomycin dosing the therapeutic target of AUC/MIC > 400 is achieved only by 25% of neonates. Appropriate PKPD targets and respective dosing regimens need to be defined in prospective clinical studies in this population.

## What is already known about this subject?

1. Vancomycin PK has wide inter-individual variability, especially in neonates
2. AUC/MIC > 400 is associated with improved treatment efficacy in adult MRSA pneumonia
3. AUC/MIC > 400 is hardly achieved with currently recommended regimes in children

## What this study adds?

1. Trough concentrations of vancomycin required for AUC 400 in neonates are lower than reported for adults
2. Based on actual MIC distribution of neonatal invasive CoNS strains, with current vancomycin dosing AUC/MIC of 400 is achieved in less than half of neonates. Vancomycin doses required for 80% probability of this target attainment are associated with potentially toxic trough levels.
3. With current vancomycin dosing, treatment failure occurs in about a quarter of cases.

## Background

With few exceptions coagulase-negative staphylococci (CoNS) have remained the most frequent causative agents of neonatal late onset sepsis over the last decades [1, 2]. Although CoNS sepsis is characterised by low mortality, the microorganism is known to be highly resistant to antibiotics - methicillin resistance exceeds 70% in most centres [3]. Therefore vancomycin has become the antimicrobial therapy of choice for targeted or empiric treatment of neonatal sepsis, especially in extremely premature babies [4]. However, despite extensive research and widespread use considerable controversies in vancomycin exposure required for optimal efficacy and consequently in dosing, remain [5, 6]. Poor tissue penetration of vancomycin suggests that different serum concentrations may be required depending on the site of infection, i.e. pneumonia vs blood stream vs bone infection [7]. Further complexity is added by pathogen-specific pharmacokinetic/pharmacodynamic (PK/PD) targets, supported by recent experimental studies. Leiva et al. have demonstrated in the neutropenic mouse thigh infection model without the use of a foreign body that to stop or kill methicillin-resistant S. *epidermidis* 2.5–3.0 times lower area under the curve to minimal inhibitory concentration ratios (AUC/MIC) were needed compared to values required for methicillin-susceptible S. *aureus* [8].

For neonates, in whom CoNS blood stream infections (BSI) predominate, effective vancomycin AUC/MIC has not been studied, but lower ratios could likely be sufficient as CoNS rarely invades tissues where vancomycin concentrations are lower than in blood.

Current vancomycin treatment in neonates is guided by the trough concentration (C_trough_) values derived from studies in adults and recommendations range between 5 and 20 mg/L. Modelling studies have shown that a C_trough_ of at least 15 mg/L is required to achieve an AUC/MIC >400 for a pathogen with MIC of 1 mg/L in adults, but lower values of 8–9 mg/L for children and 7–11 mg/L for neonates have been suggested in recent studies using samples, collected for therapeutic drug monitoring (TDM), and population pharmacokinetic (popPK) analysis [5, 9–11]. Vancomycin C_trough_ values used regularly in clinical practice unfortunately poorly predict AUC [12, 13]. Furthermore, total vancomycin concentrations are usually measured, but only unbound fraction of drug is active. Free vancomycin concentration cannot be predicted from total vancomycin concentration measurement [14].

We assumed that compared to S. *aureus* infection in adults, lower AUC/MIC ratio is needed for the treatment of neonatal CoNS sepsis because CoNS are less invasive and rarely cause other infection than BSI. In addition higher free drug concentration can be assumed in neonates due to lower protein levels and drug protein binding capability of neonatal plasma and elevated bilirubin which competes with drugs for binding sites to albumin [15–17]. Finally in our study we used MIC values of CoNS causing neonatal sepsis rather than fixed MIC values from databases.

We aimed, first, to identify neonatal vancomycin doses needed for 80% and 90% probability of target attainment (PTA) of AUC/MIC >400 and AUC/MIC >300 using minimal inhibitory concentration (MIC) distribution of invasive neonatal CoNS isolates from our hospital; second, to characterise the PTA for these targets with currently recommended TDM guided dosing, and third, to correlate individual AUC/MIC ratios with clinical outcome.

## Methods

### Study design

The clinical and laboratory data were collected retrospectively from hospital records and vancomycin concentrations from laboratory databases. Microsoft Excel spreadsheets were used to create the study database. Neonates and infants <90 days of postnatal age (PNA) admitted to Tartu University Hospital between January 1, 2010 and December 31, 2012 were included.

All neonates/infants with at least one vancomycin concentration in the database were eligible. The following data were collected: demographic parameters (gestational age [GA] and PNA, postmenstrual age (PMA), birth- and current weight [BW and CW], gender), therapeutic interventions during vancomycin treatment (mode of ventilation, presence and removal of indwelling catheters and vasoactive treatment), creatinine concentrations (measured by Jaffe kinetic method) at ±72 h of TDM time and reasons for vancomycin treatment, classified as culture proven Gram-positive sepsis, clinical sepsis and no Gram-positive sepsis [18]. Sepsis was considered culture proven if the patient had a Gram-positive pathogen isolated from blood and vancomycin treatment was given for at least 72 h; and clinical sepsis if blood culture was negative but the patient had clinical signs/symptoms suggestive of sepsis and vancomycin treatment was given for at least 72 h. All remaining cases were categorised as no Gram-positive sepsis.

### Vancomycin dosing and TDM

**V**ancomycin was given as a 60 min infusion at the dose recommended in Neofax (Table 1) [19]. Individual dose adjustments based on the TDM results were performed at the discretion of the treating physician. Start and termination of vancomycin therapy, dosing regimen and infusion time prior to TDM sample and TDM sampling time were collected.

**Table 1:** Vancomycin dosing regimen as recommended by Neofax 2010 and used in this study

PMA (week)	PNA (day)	Interval (hour)	Daily dose (mg/kg/24 h)
≤29	0–14	18	13
	>14	12	20
30–36	0–14	12	20
	>14	8	30
37–44	0–7	12	20
	>7	8	30
≥45	all	6	40

In our hospital vancomycin TDM samples are routinely taken an hour before the 3^rd^ or 4^th^ dose and thereafter at the discretion of the treating physician. Vancomycin concentrations were measured by commercial fluorescence polarization immunoassay according to manufacturers’ instructions (Cobas Integra 400/800 Analyzer, Roche, Mannheim, Germany). TDM samples taken up to 2 h before the next dose were designated C_troughs_.

### Microbiological data

Of all Gram-positive isolates obtained from blood cultures up to 5 days before and/or during vancomycin treatment of study patients were identified from the databases of the Department of Clinical Microbiology. Microorganisms were identified by the VITEK 2 microbial identification system, (bioMérieux, Lyon, France). MICs were determined using E-tests (AB Biodisk, Sweden and Liofilchem, Italy). The interpretative criteria recommended by the CLSI (2010) and by EUCAST (2011, 2012) were applied.

Additionally, vancomycin MIC data of all CoNS strains isolated from blood cultures of patients aged <90 days, admitted to our hospital during the study period, were extracted from the database of the Clinical Microbiology Department.

### Assessment of late onset sepsis (LOS) outcome

Outcome was assessed in patients with culture proven sepsis and categorised as follows: (1) eradication – negative culture within 72 h of vancomycin treatment and clinical improvement (stabilization of hemodynamics and resolution of respiratory distress symptoms); (2) presumed eradication – repeat blood culture not taken within 72 h of vancomycin treatment and clinical improvement; (3) persistence – phenotypically similar microorganisms isolated from blood after 72 h of vancomycin treatment; (4) presumed persistence – blood culture not taken and no clinical improvement or worsening of clinical condition; (5) new infection – phenotypically different CoNS spp. in blood culture after at least 72 h of vancomycin treatment; (6) relapse – positive blood culture with phenotypically similar microorganism more than 96 h after completion of vancomycin treatment. In the PK/PD analysis the first two categories were classified as success and all others as failure, including one death.

### PK/PD analysis

A two-step approach as described below was employed

### Anderson population model

Population PK analysis with 1-compartment linear model, zero-order input (1 h intravenous infusion) and 1-order elimination as described by Anderson et al. was used to estimate population parameters for clearance (Cl) and volume of distribution (Vd) [20]. Cl was standardised to a 70-kg person using allometric scaling (Fal = [Wt/70]^0.75^). Hill equation was used to estimate the maturation of Cl in Anderson model: FPMA = PMA**^**(HillCl)/(PMA**^**HillCl + EMATCl_50**^**HillCl). Renal function (RF) standardized to a 70-kg person was estimated as a component of creatinine production rate (CPR = 516 × exp(K_age × [(PMA − 40)/12]) μmol h − 1) and serum creatinine. Serum creatinine values, mean ± SD assuming a log-normal distribution, were fixed according to PMA and were based on previously published data [21]. Simulations showed that when applying renal function component as described by Anderson et al. the Cl values were positively skewed [20]. Thus, simulation procedure the RF component was fixed as 1 to avoid distorting the values.

Vd was standardised to a 70-kg person using allometric coefficient 1 (Fal = Wt/70). In the Anderson model, significant covariates were weight and PMA, hence, Cl and Vd was generated for eight PMA groups with respective weight categories. Significant covariates were continuous positive airway pressure (CPAP) ventilation for Cl, increasing clearance by 3%, and the use of inotropes for Vd, increasing Vd by 19% [20].

### TDM population model

A two-compartmental population PK model parameterized by Cl, intercompartmental clearance (Q), distribution volume in central compartment (Vc) and distribution volume in peripheral compartment (Vp) was created. Bayesian posterior distribution of model parameters were estimated using nonparametric adaptive grid algorithm for study patients based only on their TDM values [22].

### Simulations of target attainment with current dosing

Simulations were carried out using the above described models. First, Anderson's popPK model was used with collected subjects’ covariate information for retrieval of individual PK parameters. Individual AUC values were evaluated from: AUC = (24*D)/(CL*tau), where tau equals the interval between infusions (h). Second, TDM population model based on individual Bayesian posterior predictions were used and fitted concentration curves were constructed for each subject. Twenty four hour individual AUCs at steady state were calculated using the trapezoidal approximation method of fitted concentration curves.

1000 virtual MIC values were generated by bootstrapping vancomycin MICs of CoNS isolates in our database. A discrete distribution of variables was assumed. AUC values obtained by both methods were bootstrapped to 1000 virtual subjects along with MIC values and 500 such data sets were simulated for AUC/MIC targets. PTA values of 80% and 90% were found over target levels of AUC/MIC > 400 and AUC/MIC > 300. The higher target was chosen as being associated with clinical efficacy in adult MRSA pneumonia and the most frequently quoted also in neonatal PKPD simulation studies in recent years [23]. A lower target was also tested, as different pathogen structure and disease (blood stream vs lung tissue penetration) as well as lower protein binding suggest lower optimal exposure for neonates. Empirical confidence intervals were identified from performed simulations.

### Calculation of vancomycin doses needed for therapeutic target attainment

Using Monte Carlo simulations (MCS), vancomycin doses needed to achieve 80% and 90% PTA of AUC/MIC > 400 and AUC/MIC >300 were estimated for 1000 virtual subjects. Vancomycin MIC distribution of CoNS isolates from our database and fixed MIC values of 1, 2 and 4 mg/L were used as base levels for target simulations. The Cl and Vd derived from the Anderson population model, predicted with and without CPAP ventilation and inotropes, were used to calculate individual AUC and vancomycin MIC values of CoNS isolates. AUC and MIC values were bootstrapped assuming discrete distribution. Simulated vancomycin doses needed to achieve 90% and 80% PTA of AUC/MIC >400 and AUC/MIC >300 and model predicted Cl for each PMA group were used to calculate corresponding vancomycin maximum concentrations (C_max_) and C_troughs_ at steady state.

### Correlation of AUC/MIC with outcome

Dosing data and C_trough_ concentrations of the study population were used to estimate individual AUC/MIC values in subjects with positive blood cultures. Multiple LOS episodes were handled as separate cases if at least 96 h interval between vancomycin treatment episodes was present. Individual AUC/MIC estimates were obtained matching specific case-wise averaged AUC values with the corresponding MIC values. In case of mixed infections AUC/MIC values were obtained for each isolate separately.

All calculations were performed with the R Project for Statistical Computing (version 2.15.2 (2012-10-26), The R Foundation for Statistical Computing) and Pmetrics package [22, 24]. Comparison of PK characteristics was done with Mann–Whitney-Wilcoxon test and outcome association to AUC/MIC with Kruskal-Wallis chi-squared test.

The protocol was approved by the Ethics Committee of the University of Tartu. Informed consent was not needed due to retrospective nature of the study.

## Results

### Study population

In total, of 201 vancomycin TDM measurements from 80 eligible patients were registered in the laboratory database. Medical records of four patients were not available and they were excluded. The final dataset included 76 patients with 88 vancomycin treatment episodes and 186 TDM data points; in 83 episodes vancomycin was given for more than 72 h. In 5 cases, one treated for suspected meningitis, three without confirmed infection and two with Gram-negative infection, vancomycin treatment was discontinued. Among vancomycin treated patients 57 episodes were culture proven, seven with two different Gram-positive microorganisms.

Demographic parameters of the study population are shown in Table 2. Overall 39/57 patients with proven LOS had successful outcome (eradication – 37, presumed eradication – 2) and 18/57 were classified as failures (persistence – 10, presumed persistence – 1, new infection – 5, relapse – 2).

**Table 2:** Demographic and clinical data at the time of TDM measurement

	Culture proven sepsis	All
Male/female (n)	26/20	44/32
GA (weeks) mean ± SD	27.4 ± 3.3	28.9 ± 4.2
BW (g) mean ± SD	1100.0 ± 604.7	1346.9 ± 810.7
CW (g) mean ± SD	1104.2 ± 580.8	1316.8 ± 843.1
Patients (n)/TDM samples (n)	46/142	76/187
PMA (weeks) mean ± SD at first TDM	29.3 ± 4.1	30.9 ± 4.8
PNA (days) mean ± SD at first TDM	11 ± 11	12 ± 11
Mean creatinine concentration (μmol/L)	53 ± 23	51 ± 22
CPAP (%)	23	25
Mechanical ventilation (% of patients)	60	58
Vasoactive treatment (% of patients)	44	46
Vancomycin treatment duration (days)	10 ± 4	11 ± 4
LOS (n of episodes)	57	83
Died (n)	1	3

### Microorganisms

**Fig. 1:**
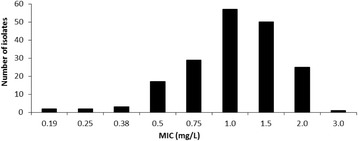
Distribution of vancomycin MIC values of all neonatal blood CoNS isolates from the Tartu University Hospital in 2010–2012

Vancomycin MIC distribution of all 187 neonatal CoNS blood isolates is shown in Fig. 1. The median MIC value was 1.0 mg/L (range 0.2–3.0; IQR, 0.8–1.5); 59% of isolates had a MIC ≤1.0 mg/L. Among study patients 99/104 blood cultures were positive for CoNS (*S. haemolyticus* 38*, S. epidermidis* 37*, S. hominis* 12*, S. warneri 8, S. capitis* 1*, S. saprophyticus* 2, S. *lentus* 1), 4/104 for *E. faecalis* and *1/104* for *S. parasanguis.* Their median MIC value was 1.0 mg/L (range 0.5–3.0); similar to the entire CoNS dataset.

### PK characteristics of the study population

Vancomycin TDM measurements were taken after the 1^st^ to 44^th^ dose (median 5; IQR 3–8) between 4.5 h and 24 h after the start of infusion. The median number of TDMs per patient was 2 (range 1–9); C_trough_ of 5–10 mg/L was achieved in 63.6% and 10–15 mg/L in 20.5% of cases. There were 9.3% and 7.3% samples with vancomycin concentration below 5 mg/L and above 15 mg/L, respectively.

PK parameters (shown as mean ± SD) obtained from the Anderson model were Vd = 0.61 ± 0.05 L/kg; Cl = 0.06 ± 0.01 L/h*kg and parameters obtained from the popPK model were Vc = 0.13 ± 0.06 L/kg; Cl = 0.06 ± 0.02 L/h*kg; Q = 0.36 ± 0.10 L/h*kg; Vp = 0.76 ± 0.22 L/kg.

Mean (SD) vancomycin AUC values obtained in TDM and the Anderson model were similar 280.6 ± 102.0 mg/kg*L and 287.1 ± 74.2 mg/kg*L, respectively. AUC values throughout the studied GA range were similar (data not shown).

### Clinical outcome vs AUC/MIC in subjects with culture proven sepsis

**Fig. 2:**
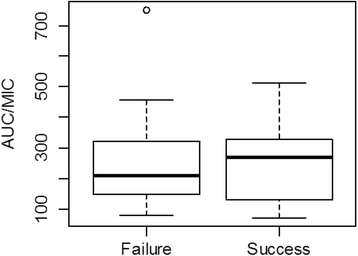
Boxplot of TDM based vancomycin AUC/MIC and clinical outcome of culture proven LOS. Black bold line is median and whiskers show lower and upper quartiles

Only 34% and 12% of subjects with positive blood cultures achieved AUC/MIC ≥300 and AUC/MIC ≥400, respectively. Clinical outcome and individual AUC/MIC values of culture positive LOS episodes are shown in Fig. 2. The AUC/MIC ratio between patients with clinical success (median 268.6; IQR 130.7–326.5) and failure (median 210.1; IQR 149.5–319.1) was similar.

### PTA in study population

**Fig. 3:**
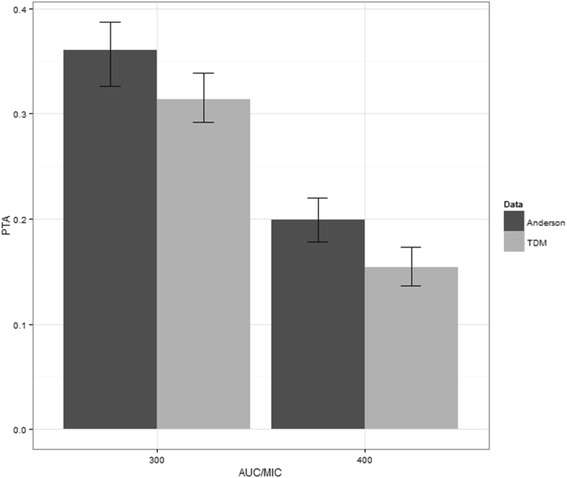
90% PTA with 95% confidence intervals (error bars) with the current dosing for MIC distribution of neonatal blood isolates in the study group, AUC calculation was based on TDM and Anderson model [19]

MCS results of 90% PTA in the whole study population with current dosing are presented in Fig. 3. Target of AUC/MIC >300 was achieved in less than 40% and AUC/MIC >400 in less than 25% of subjects regardless of used calculation method.

### Vancomycin doses required for 80% PTA

Simulated daily doses of vancomycin required for 80% PTA by PMA band are shown in Tables 3 and 4, respectively. Since only 25% of TDM patients were receiving CPAP and 46% were supported with inotropes, only data of non-CPAP with the use of inotropes are presented. For 80% PTA of AUC/MIC >300 the calculated doses were similar to those in NeoFax up to MIC of 2.0 mg/L, inclusive. The corresponding C_trough_ values remained ≤20 mg/L, whereas for MIC of 4.0 mg/L the corresponding vancomycin C_trough_ levels ranged between 21.8–31.6 and 28.7–41.0 mg/L for AUC/MIC >300 and AUC/MC >400, respectively. Mean actually used vancomycin doses (data not shown) were below those required for 80% PTA of AUC/MIC >300 and AUC/MIC >400 for MIC of ≥2.0 mg/L and for AUC/MIC >400 for MIC distribution of all neonatal blood CoNS isolates from our hospital.

**Table 3:** Monte Carlo simulations of vancomycin doses needed for 80% probability of target attainment of AUC/MIC > 300 at MIC distribution of CoNS isolates and at fixed MIC values

PMA	MIC 4		MIC 2		MIC 1		Hospital MIC	
	Dose	C_tough_	Dose	C_trough_	Dose	C_trough_	Dose	C_trough_
24	25.0	31.6	14.0	17.7	8.0	10.1	11.5	14.6
25	26.5	30.4	15.0	17.2	8.5	9.7	12.0	13.8
26	27.0	29.5	16.0	17.4	9.0	9.8	12.5	13.6
27	29.0	28.6	16.5	16.3	9.5	9.3	13.0	12.8
28	30.5	27.2	18.0	16.1	10.5	9.4	14.0	12.5
29	33.0	27.3	19.0	15.7	11.0	9.1	14.5	12.0
30	34.0	26.1	19.5	15.0	11.0	8.5	15.5	11.9
34	37.0	24.3	21.0	13.7	12.5	8.2	17.0	11.1
40	43.5	21.8	25.0	12.2	14.5	7.1	19.5	9.5

PMA (postmenstrual age in weeks)

C_trough_ (steady state vancomycin through plasma concentration in mg/L)

Dose (mean administered vancomycin dose in mg/kg/24 h)

MIC (fixed vancomycin MIC value in mg/L)

Hospital MIC (vancomycin MIC distribution of CoNS isolates in our hospital in mg/L)

**Table 4:** Monte Carlo simulations of vancomycin doses needed for 80% probability of target attainment of AUC/MIC > 400 at MIC distribution of CoNS isolates and at fixed MIC values

PMA	MIC 4		MIC 2		MIC 1		Hospital MIC	
	Dose	C_trough_	Dose	C_trough_	Dose	C_trough_	Dose	C_trough_
24	32.5	41.0	18.5	23.4	11.0	13.9	14.5	18.4
25	35.5	40.6	20.0	22.9	11.5	13.2	16.0	18.3
26	36.0	39.2	20.5	22.4	12.0	13.1	16.5	18.0
27	38.5	37.8	22.0	21.7	13.0	12.8	17.5	17.2
28	41.0	36.6	23.5	21.0	13.5	12.0	18.5	16.5
29	44.0	36.3	24.5	20.2	14.0	11.6	19.5	16.1
30	44.5	34.2	25.5	19.6	15.0	11.5	20.5	15.8
34	49.5	32.3	28.0	18.3	16.5	10.9	22.5	14.8
40	58.5	28.7	33.5	16.4	19.0	9.3	26.5	13.0

PMA (postmenstrual age in weeks)

Ctrough (steady state vancomycin through plasma concentration in mg/L)

Dose (mean administered vancomycin dose in mg/kg/24 h)

MIC (fixed vancomycin MIC value in mg/L)

Hospital MIC (vancomycin MIC distribution of CoNS isolates in our hospital in mg/L)

## Discussion

Based on vancomycin TDM results and actual MIC values of neonatal CoNS isolates we showed that with currently recommended NeoFax dosing regimen the 80% PTA of AUC/MIC >400 and AUC/MIC >300 is achievable only in less than half of infected neonates. Furthermore, targeting less susceptible organisms (MIC values of >2.0 mg/L) achievement of 80% PTA of AUC/MIC >400 would require the use of much higher vancomycin doses that result in C_trough_ levels well above 20 mg/L. These results suggest that current dosing recommendations in NeoFax [and likely also in British National Formulary (BNF)] may not be adequate for treatment of neonatal CoNS sepsis. The same conclusion was reached by authors who studied vancomycin C_troughs_ achieved by neonates and compared these to vancomycin MICs of CoNS isolates [25].

However, when interpreting these results several outstanding issues remain before firm conclusions can be drawn for everyday practice. First, the most appropriate AUC/MIC ratio for treatment of CoNS infections that are predominantly BSI rather than deep-sided infections has been defined neither in neonates nor in adults. In a recent study Ramos-Martín et al. used hollow fibre infection model (HFIM) and a novel rabbit model of neonatal central-line associated blood stream infections (CLABSI) and bridged these data to neonates using popPK techniques and MCS. Both experimental models suggested higher AUC/MIC targets for neonates (665 for the HFIM and 520 for the CLABSI model) than are currently proposed for adult MRSA infection to achieve maximal killing, prevent emergence of a resistant subpopulation and suppress C-reactive protein in the setting of a retained central line [26]. Whether such high levels are required or can be safely used in clinical settings for every (premature) neonate if central line is removed or if vancomycin is given empirically or if clinical rather than culture proven LOS is managed, is still debatable and will be tested in a clinical study before recommendation for clinical practice can be made [27]. In contrast, other experimental data of thigh infection in mice suggest that the required AUC/MIC ratio for treatment of CoNS infection is about 2.5 to 3 times lower than that required for treatment of S.*aureus* infection [8]. Moreover, higher free drug concentration can be assumed in neonates due to lower drug protein binding capacity of neonatal plasma and elevated bilirubin which competes with drugs for binding sites to albumin [15–17].

Our findings that there was no association between AUC/MIC ratio and outcome do not prove that such association does not exist. First, one should bear in mind these data are retrospectively collected and thus outcome measures may be prone to bias. Second, it may well be that AUC/MIC ratio of 300 and 400 assessed by us are too similar to detect difference between these two ratios. We believe that more data with a larger dataset in clinical CoNS infections are needed in terms of optimal AUC/MIC ratio before firm conclusions on adequacy of current dosing recommendations can be drawn.

We and others have shown that to achieve AUC/MIC value of 400 the vancomycin trough levels around 10 mg/L or slightly higher are required provided that MIC value of an infecting organisms is ≤1 mcg/ml. The latter was the median value of Gram positive isolates in our hospital as well. If however, the distribution of actual isolates was used in simulations it appeared that only approximately 35% of neonates achieved AUC/MIC values of 300 and less than 22% of 400 suggesting that these targets are not achieved with current dosing in the majority of patients. However, one should emphasize once again that we still do not know whether these targets are required at all.

We have recently shown that in fact 75% of vancomycin doses used in European NICUs are lower than recommended by BNF (and also by NeoFax) [28]. We speculated that one reason for that might a fear of toxicity by neonatologists but acknowledge that this was not specifically studied by us. Association between vancomycin C_trough_ and risk of nephrotoxicity as a function of intensity and duration of therapy, compounded by additional risk factors, is suggested but data in the literature are too inconsistent to demonstrate direct causal relationship with kidney or to define certain nephrotoxic levels for neonates [29, 30]. Steady-state concentration of ≥28 mg/L has been associated with increased risk of renal toxicity in patients treated with continuous infusions [31]. Studies with intermittent treatment have suggested C_trough_ level >15 mg/L for increased risk of nephrotoxicity in adults and children [32, 33]. Applying C_trough_ 15 mg/L as nephrotoxic breakpoint all doses needed for achievement of AUC/MIC >300 and AUC/MIC >400 for pathogens with MIC ≥2.0 mg/L resulted in nephrotoxic values or remained slightly lower in more mature neonates. Studies evaluating long term effects of vancomycin therapy on hearing in neonates are almost entirely missing [34, 35]. Thus far there is no indication on vancomycin ototoxicity [36]. Such studies are complex and should include control group as several neonatal conditions (e.g. hypoxia, sepsis, bronchopulmonary dysplasia, genetic abnormalities etc.) have been associated with hearing disturbances [37].

Although numerous studies on vancomycin PK using various models and popPK approach have been published almost all of them including our study are based on the samples collected for TDM purposes in everyday practice and are thus prone to high variability in sample collection and used laboratory methods; all this affecting final results [38, 39]. Prospectively designed studies with rich or semi-rich sampling are scarce or outdated in neonates [40]. At present NeoVanc project sponsored by EU FP7 is aiming to fill these gaps. The project aims to define the most optimal PD target using in vitro hollow fibre and in vivo rabbit model of CoNS sepsis. The preclinical data will be validated in the multicentre randomised controlled trial comparing efficacy and safety of standard to optimised vancomycin dosing regimen in neonates and infants with sepsis caused by CoNS. The long term effects of vancomycin blood concentrations on renal and hearing function will be evaluated as well [27].

Several limitations of the study need to be noted. The relatively small number of participants in each GA band may have under- or overestimated the within group variability. Due to the retrospective nature only C_trough_ data were available, as monitoring of peak concentration has been found of no value in guiding vancomycin therapy [9]. Concomitant medications and underlying illness may have influenced PK of vancomycin and minor fluctuations may have occurred in recorded C_trough_ collection time. Microbiological outcome may have been affected by combination of antibacterial drugs used in the beginning of the treatment episode.

## Conclusions

Currently recommended dosing regimens are not appropriate to achieve vancomycin PK/PD target of AUC/MIC >400, as recommended for adult MRSA infection, or even AUC/MIC >300, since the target is reached in less than 40% of neonates with sepsis. Adequately designed preclinical and clinical studies should first establish optimal PK/PD targets for CoNS BSI, second define optimal dosing regimen to achieve these targets for premature neonates and third to identify prevalence and risk factors of vancomycin induced nephro- and ototoxicity among neonates in long term follow up studies. When making recommendations for treatment of CoNS infection in neonates appropriate balance between relatively benign outcome and potential toxicity should be weighted.

## Abbreviations

AUC/MIC: Area under the curve to minimal inhibitory concentration ratio
BNF: British national formulary
BSI: Blood stream infection
BW: Birth weight
Cl: Clearance
CLABSI: Central-line associated blood stream infection
CLSI: The clinical & laboratory standards institute
C_max_: Maximum concentration
CoNS: Coagulase-negative staphylococcus
CPAP: Continuous positive airway pressure
CPR: Creatinine production rate
C_trough_: Trough concentration
CW: Current weight
EUCAST: The European committee on antimicrobial susceptibility testing
FP7: Seventh framework programme for research and technological development
GA: Gestational age
HFIM: Hollow fibre infection model
LOS: Late onset sepsis
MCS: Monte Carlo simulation
MRSA: Methicillin-resistant staphylococcus aureus
PKPD: Pharmacokinetic-pharmacodynamic
PMA: Postmenstrual age
PNA: Postnatal age
PopPK: Population pharmacokinetics
PTA: Probability of the target
Q: Intercompartmental clearance
RF: Renal function
TDM: Therapeutic drug monitoring
Vc: Distribution volume in central compartment
Vd: Volume of distribution
Vp: Distribution volume in peripheral compartment.
